# Review of the cardiovascular safety of COXIBs compared to NSAIDS

**Published:** 2008-04

**Authors:** I Moodley

**Affiliations:** Health Outcomes Research Unit, Department of Public Health and Family Medicine, Nelson R Mandela School of Medicine, Durban

## Abstract

**Summary:**

There is no doubt that NSAIDs and COXIBS are the mainstay for managing pain and inflammation in arthritis. Overall, at therapeutically equivalent doses, both NSAIDs and COXIBs provide equivalent analgesic and anti-inflammatory efficacy. However, the gastrointestinal risk associated with NSAIDs is considerable. More recently, the cardiovascular risk associated with NSAIDs and COXIBs has become a concern.

Most patients, particularly the young, can benefit from NSAIDs without the risk of serious adverse gastrointestinal or cardiovascular events. However, patients with a previous history of serious gastrointestinal complications and the elderly, who could be at risk, do require alternatives.

COXIBs have significant benefits over NSAIDs in reducing the incidence of serious gastrointestinal complications (perforations, ulcers and gastric bleeding). Currently two oral COXIBs are available, celecoxib and lumiracoxib, and one parenteral COXIB, parecoxib. Celecoxib has been on the market for longer and has the largest body of evidence.

The older NSAIDs, such as meloxicam, with preferential COX-2 inhibition do not have good long-term evidence of reducing the incidence of serious gastrointestinal complications. However, these agents do have evidence of tolerability, ie, reducing the less-serious gastrointestinal effects, mainly dyspepsia. The South African Rheumatoid Arthritis Association’s guidelines, amended in November 2005 recommend COXIBs for elderly patients (> 60 years) with previous gastropathy and those on warfarin and/or corticosteroids, providing they do not have contra-indications.

However, caution is advised when prescribing COXIBs for patients with risk factors for heart disease. These recommendations are very similar to those made by the National Institute for Clinical Excellence (NICE). In addition, it should be noted that for those patients without any cardiovascular complications but with gastrointestinal risk factors or on aspirin, it may be necessary to add a proton pump inhibitor (PPI). PPIs, however, provide little benefit for bleeding and ulceration of the lower intestine. One consequence of this low-grade bleeding is anaemia and a general feeling of malaise in patients with rheumatic disease. Current evidence suggests that COXIBs such as rofecoxib and celecoxib do not increase small intestinal permeability and that celecoxib does not cause lower intestinal bleeding and may be of benefit to those patients with lower gastrointestinal complications.

In patients at risk for cardiovascular complications, both NSAIDs and COXIBs have been shown to increase the risk of myocardial infarctions (MI), hypertension and heart failure. Studies comparing COXIBs and non-specific NSAIDs should, however, be interpreted with caution. One needs to take into account the underlying baseline cardiovascular risk of the populations being compared. COXIBs appear to be prescribed preferentially to patients who were at an increased risk of cardiovascular events compared with patients prescribed non-specific NSAIDs.

When the overall risk of cardiovascular complications is relatively low and an anti-inflammatory agent is required, current evidence suggests that celecoxib is an agent of choice because of its lower cardiovascular toxicity potential compared to NSAIDs and other COXIBs.

## Summary

Non-steroidal anti-inflammatory drugs (NSAIDs) are the mainstay treatment for the management of pain and inflammation associated with arthritic diseases. However, their use is restricted because of the high incidence of side effects, particularly those relating to the gastrointestinal (GI) tract, renal and cardiovascular systems.

While the most troublesome adverse events, referred to as dyspepsia, affect the majority of users of NSAIDs, serious GI events such as perforation, ulceration and bleeding affect a significant proportion of users.^1^,^2^ The risk of these more-serious adverse events increases with factors such as age, a previous history of GI events and those treated with higher doses of NSAIDs, corticosteroids and aspirin. Selective cyclooxygenase-2 inhibitors (COXIBs), as opposed to NSAIDS, which inhibit both the cyclooxygenase-1 and -2 isoenzymes were introduced to reduce the increased risk of GI injuries to patients requiring relief from pain and inflammation. While these drugs did reduce the risk of GI injury, like other NSAIDs, they also appear to increase the risk of adverse cardiovascular events. This review attempts to evaluate the risks and benefits of COXIBs in relation to conventional NSAIDs, with a view to ascertain whether there is any differentiation between NSAIDs and COXIBs, as well as between COXIBs.

## Methods

A literature search was performed using the Medline database. Keywords used were: ‘the efficacy, gastrointestinal, cardiovascular and renal safety of coxibs, selective cyclooxygenase-2 inhibitors, specific cyclooxygenase-2 inhibitors and non-steroidal anti-inflammatory drugs, NSAIDs’ (MAJR). Review articles, clinical guidelines, letters and editorials were excluded. In the EMBASE database, the following search was conducted: (explode ‘Coxibs, selective cyclooxygenase-2 inhibitors, specific cyclooxygenase-2 inhibitors and non-steroidal anti-inflammatory drugs, NSAIDs’/adverse-drug-reaction, side-effect in DEM, DER, DRm, DRR) and [(EC:EMBV = CARDIOVASCULAR) OR EC:EMBV = GASTROINTESTINAL) or EC:EMBV = RENAL) or EC:EMBV = HEPATIC)].

Articles were then manually selected based on relevance and the reference sections were studied for additional ones. Other sources of literature utilised included the Cochrane Library and the websites of the European Agency for the Evaluation of Medicinal Products (EMEA) and FDA. The searches were conducted for articles published in English from 1986 to 2006.

The outcome of the literature search was to compare the efficacy and gastrointestinal, cardiovascular and renal safety of NSAIDs and COXIBs and to draw some conclusions regarding their benefits and risks for patients in need of relief of pain in inflammatory joint diseases.

## Efficacy of COXIBS and NSAIDs in osteo- and rheumatoid arthritis

It is not disputed that both NSAIDs and COXIBs are effective in providing relief from pain in inflammatory joint diseases, particularly at therapeutically equivalent doses. Some debate may revolve around small differences in efficacy between and within the different classes of drugs. A systematic review and meta-analysis of randomised placebo-controlled trials was conducted for the analgesic activity of NSAIDs and COXIBs in patients with osteoarthritis of the knee.^3^ The pooled results of COXIBs and NSAIDs, including diclofenac, naproxen, nabumetone, meloxicam, rofecoxib, etoricoxib, valdecoxib and celecoxib, for pain relief on the analogue scale was 15.6% better than placebo.

While it is evident that NSAIDs and COXIBs are significantly more effective than placebo, some variation was seen in efficacy between the different drugs. For example, in the 28-day MELISSA study, there was a trend favouring diclofenac 100 mg SR over meloxicam 7.5 mg, with more patients discontinuing meloxicam due to lack of efficacy.^4^ Similar findings were observed in patients with rheumatoid arthritis,^5^ with naproxen 750 mg showing superior efficacy to meloxicam 7.5 mg. However, at comparable doses, meloxicam 15 mg, celecoxib 200 mg, nabumetone 100 mg or diclofenac 75−100 mg during a six-month period of treatment for rheumatoid arthritis were equi-active.^6^ In this study, nine to 12% of patients withdrew from the study due to lack of efficacy, with the exception of celecoxib, which was mainly for reasons of higher cost.

Overall, it can be concluded that at therapeutically equivalent doses, both NSAIDs and COXIBs provide equivalent analgesic and anti-inflammatory efficacy. However, it is recognised that NSAIDs and COXIBs may not be used on a continuous basis as analgesic and anti-inflammatory drugs because of the risk of serious adverse events.^3^

## Gastrointestinal safety of COXIBs and NSAIDs

A number of studies have compared the GI safety of non-selective NSAIDs and COXIBs, with equivocal findings. Direct comparisons are often not possible for a host of reasons. These include differing study designs, comparators, doses and outcome measures, among others. The outcome measures vary across the studies, ranging in severity from mild symptoms such as GI tolerability or dyspepsia to more severe conditions such as GI bleeding, ulcers and perforation. An attempt will be made to rationalise studies with comparable outcomes so that some meaningful conclusions can be drawn.

For conventional NSAIDs and COXIBs, there are few longterm randomised, double-blind GI safety studies. The longest randomised studies available are for rofecoxib, celecoxib and etodolac. Short-term studies are available for meloxicam.

The overall evidence suggests that in longer-term safety studies, celecoxib demonstrates fewer serious adverse GI events and better gastric tolerability than other NSAIDs. However, it may be worth noting that there is no evidence of a direct association between the incidence of gastric intolerability and gastrointestinal ulcers and more serious gastrointestinal events.

Apart from their adverse effects on the upper GI, a number of studies suggest that NSAIDs are implicated in lower GI tract injury and complications.^7^ In the general population, lower GI tract complications such as bleeding occur at a rate equaling approximately one-fifth the rate of upper GI tract complications. 2,8 The long-term effects on the lower gut due to chronic NSAID use at any dose are associated with anaemia and inflammation. 9,10

However, the lower GI effects of NSAIDs have been less extensively studied and characterised than the upper GI tract effects. Observational studies have shown that the relative risk increase of lower GI tract complications with NSAIDs is comparable with the relative risk increase of upper GI events.^11^-^13^ Current evidence suggests that COXIBs such as rofecoxib and celecoxib do not increase small intestinal permeability^14^,^15^ and that COXIBs may offer additionally reduced risk of lower GI injury.

## Cardiovascular safety of COXIBS and NSAIDs

Serious concern was raised with reports of increased deaths associated with COXIBs and in particular, rofecoxib. This concern once again highlighted the risk of cardiovascular complications that were known with NSAIDs, now extending to COXIBs. An increased risk of acute myocardial infarction has been long associated with the use of NSAIDs, such as naproxen and diclofenac, particularly in high-risk patients over the age of 50 years.^16^

The first signs of concern regarding the cardiovascular safety of COXIBs arose because of the unanticipated but significantly higher incidence of cardiovascular thrombotic events with rofecoxib compared to naproxen in the VIGOR study.^17^ In this study, patients taking rofecoxib 50 mg had a five-fold increased risk of MI compared with naproxen 1 000 mg. This observation was confirmed in the APPROVE study, which evaluated the incidence of adenomatous polyps in patients treated with rofecoxib 25 mg compared with placebo.^18^ This study showed a two-fold increase in MI risk with rofecoxib. Similar observations were reported for other more selective COXIBs.

In studies for post-operative pain relief following coronary bypass surgery with parecoxib and valdecoxib, a significantly higher risk of MI was observed in the treatment group when compared to placebo.^19^ As a consequence of these observations, both rofecoxib and valdecoxib were voluntarily withdrawn worldwide. In contrast, no significant differences in MI rates were observed with celecoxib compared to diclofenac or ibuprofen in the CLASS study^20^ and in patients with osteoarthritis in the SUCCESS-I study.^21^ However, the concern of an increased risk of cardiovascular events remains, particularly following the withdrawal of rofecoxib after a number of studies^22^ confirmed the finding of the VIGOR study.^17^

The comparative risk of MI in patients taking COXIBs and NSAIDs was assessed in 9 218 cases with a first-ever diagnosis of MI between 2000 and 2004 in a primary-care setting in the United Kingdom.^23^ The study showed that that there was a significantly increased risk of MI, particularly with rofecoxib (OR: 1.32), ibuprofen (OR: 1.24) and diclofenac (OR: 1.55) compared with no use within the previous three years. Use of other selective and non-selective NSAIDs, including naproxen also showed a significantly increased risk of MI but the magnitude was reduced after adjustment for potential confounders. No significant increase in cardiovascular risk was associated with the use of celecoxib

With respect to the newer COXIBs, in a one-year follow-up study, lumiracoxib^24^ showed a higher but statistically non-significant increased risk of MI compared with naproxen, while etoricoxib^25^ showed a similar risk for MI when compared with diclofenac.

It is evident that there is a need to specifically evaluate the cardiovascular risk of patients on NSAIDs and COXIBs, particularly in instances where there may be a means to differentiate these agents. While the cardiovascular risks associated with rofecoxib and valdecoxib were apparent, those associated with celecoxib are ambiguous. In the 12-week SUCCESS-1 study, the incidence of cardiovascular events, including hypertension, coronary artery disease, stroke and transient ischaemic attacks were not only low but similar across diclofenac, naproxen and celecoxib.^21^ A *post-hoc* analysis of the results showed a higher but non-significant difference in the incidence of MI in patients treated with celecoxib 100 mg twice a day. However, the trend did not appear dose-related as the incidence of MI in the celecoxib 200 mg twice-daily group was lower. Since the overall incidence of MI was relatively low, no robust conclusions could be drawn.

One way of trying to make sense of these observations is to pool the results in a meta-analysis. A pooled analysis of several trials^26^ has shown no increase in cardiovascular events with celecoxib. However, at higher doses, studies have shown a higher risk of MI with celecoxib. In evaluating celecoxib to prevent colon polyps (treatment duration 2.8−3.1 years), patients treated with celecoxib 200 and 400 mg twice daily had a 2.3 and 3.4 times greater risk of cardiovascular events than placebo.^27^ Contrasting observations were made in a similar study where celecoxib 400 mg once daily did not show any significant increase in cardiovascular events compared with placebo.^28^ The Alzheimer’s Disease Anti-inflammatory Prevention Trial (ADAPT) again showed no significant increase in cardiovascular events with celecoxib compared with placebo, in contrast to naproxen where a significant increase in cardiovascular events compared to placebo was observed.^29^

While it is possible that the potential for increased risk of MI may be associated with long-term sustained suppression of COX-2, particularly at the higher doses of celecoxib (400 mg twice daily), shorter-term therapeutic doses may have an anti-inflammatory effect on endothelial function. Patients with coronary artery disease treated for two weeks with celecoxib 200 mg twice daily showed an improvement in endothelial function and reduced high-sensitivity C-reactive protein and oxidised low-density lipoprotein.^30^

A nested case-control study to evaluate risk of coronary heart disease using data from a Californian managed-care organisation was conducted in a cohort of all patients between the ages of 18 and 84 years treated with a NSAID for a one-year period.^31^ Cases of serious coronary heart disease (acute MI and sudden cardiac death) were risk-set matched with four controls for age, gender and health plan region. Current exposure to COXIBS and non-selective NSAIDs was compared with remote exposure to any NSAID, and rofecoxib was compared with celecoxib.

During the period of the analysis, 8 143 cases of serious coronary heart disease occurred, of which 2 210 (27.1%) were fatal. Multivariate adjusted odds ratios versus celecoxib were for rofecoxib (all doses), 1.59; rofecoxib 25 mg/day or less, 1.47; and rofecoxib greater than 25 mg/day, 3.58. For naproxen versus remote NSAID use, the adjusted odds ratio was 1.14. The analysis indicated that rofecoxib use increased the risk of serious coronary heart disease compared with celecoxib use, while naproxen use did protect against serious coronary heart disease.

The net cardiovascular (coronary heart disease, stroke, congestive heart failure) and GI (peptic ulcer complications) risk−benefit and public health impact of the use of celecoxib compared to non-selective NSAIDs was estimated in an arthritis population. Discrete event simulation models were applied to data from the US National Health Surveys, CV risk-prediction models from the Framingham Heart Study and population-based studies. Models took into account the multifactorial effect of risk factors, co-morbidity and competing risk of mortality, and simulated the natural history of CV and GI disease in the US arthritis population over one year, through the individual baseline cardiovascular and gastrointestinal risk profile. This model was modified with relative risks associated with the use of each treatment. The mean number of events was estimated for each endpoint in each model: natural history, celecoxib, diclofenac, ibuprofen and naproxen. The number of events for celecoxib was compared with each NSAID. The evaluation included 1% of the US population with arthritis. No increase in cardiovascular events or all-cause mortality was observed for celecoxib versus the diclofenac, ibuprofen and naproxen.^32^

## Renal safety of COXIBS and NSAIDs

It is well documented that NSAIDs may cause fluid retention and should be avoided in patients who present with severe congestive heart failure and severe hypertension.^33^ Moreover, patients with rheumatoid arthritis are at increased risk for cardiovascular complications. A random sample of a North Glasgow population^34^ showed that in this population, diastolic pressure and thrombotic variables were elevated compared to controls. Many of these kinds of patients are on NSAIDS and disease-modifying drugs, as well as concomitant drugs for hypertension. Given this background, it is useful to know the extent of these drug−drug interactions and how best to manage them.

As a consequence of their direct effects on renal function and destabilisation of blood pressure, interaction of anti-hypertensive drugs with NSAIDs exacerbates hypertension. NSAIDs and COXIBs interact with angiotensin-converting enzyme (ACE) inhibitors and β-blockers.^35^,^36^ Renal function in the elderly has been shown to be markedly impaired by diclofenac, particularly after treatment with ACE inhibitors.^37^ However, the effects of NSAIDs on cardio-renal function may vary according to the drug used. For example, indomethacin and naproxen appear to cause increases in mean arterial blood pressure in hypertensive patients, whereas such effects are less acute with sulindac, aspirin and ibuprofen.^33^,^35^

The early indications of adverse renal events caused by COXIBs were reported from the World Health Organisation’s Uppsala Monitoring Centre Safety database that compared spontaneously reported renal-related adverse drug reactions for celecoxibs and rofecoxib.^38^ It was found that both celecoxib and rofecoxib were associated with renal-related adverse drug events but the adverse renal impact of rofecoxib was significantly greater than that for celecoxib. Rofecoxib was also associated with significantly greater water retention, abnormal renal function, acute renal failure, cardiac failure and hypertension.

During early marketing, hospitalisation for acute blood pressure elevation appears to have been reported more frequently for rofecoxib compared with celecoxib, consistent with clinical trial data on file with the FDA. These and other published studies found rofecoxib has a greater effect on blood pressure than other NSAIDs, including celecoxib. This finding may be particularly relevant in older patients, given the prevalence of hypertension and cardiovascular disease in this age group. Similar findings were reported in patients over 65 years where rofecoxib significantly increased (7.37 mmHg) systolic blood pressure, whereas celecoxib caused a decrease (−1.94 mmHg). Neither drug had any effect on diastolic blood pressure. Higher incidences of hypertension and oedema were observed with 25 mg rofecoxib daily, compared with celecoxib 200 mg daily in patients over 65 years old.^36^,^39^

In a retrospective case-control study of patients aged ≥ 65 years,^40^ rofecoxib use was associated with an increased relative risk of new-onset hypertension. This risk was twice as high in those taking rofecoxib compared with celecoxib (OR 2.1; 95% CI: 1.0−4.3).

The use of NSAIDs was associated with a small increase in risk of a first hospitalisation for heart failure (HF). In patients with prior clinical diagnosis of HF, the use of NSAIDs may lead to worsening of pre-existing HF that triggers their hospital admission.^40^,^41^ This increased risk, although small, may result in considerable public health impact, particularly among the elderly.

The risk of death and recurrent congestive heart failure was compared in patients prescribed celecoxib, rofecoxib or NSAIDs in a population-based retrospective cohort study in patients over 66 years.^42^ The risk of death and recurrent congestive heart failure combined was higher in patients prescribed NSAIDs or rofexocib than in those prescribed celecoxib (hazards ratio 1.26, 95% CI: 1.00−1.57 and 1.27, 1.09−1.49, respectively). Celecoxib seems safer than rofecoxib and NSAIDs in elderly patients with congestive heart failure.

In a case-control design,^43^ nested within an administrative database cohort of patients with rheumatoid arthritis (RA) who was dispensed a disease-modifying drug or COXIB between September 1998 and December 2001, patients were assessed for risk of hospitalisation for congestive heart failure (CHF). The cohort included 41 885 patients (75% were women, with an average age at cohort entry of 51 years). During follow up, 520 hospitalisations for CHF occurred, for a rate of 10.1 per 1 000 per year.

The adjusted RR of CHF for current use of any disease-modifying drug (DMARD) was 0.7 relative to no current use. For the DMARD category, there was evidence of a beneficial effect for both tumour necrosis factor α antagonists (RR 0.5) and methotrexate monotherapy (RR 0.8). For non-DMARD medications, the rate of CHF was not clearly increased or decreased, except for COXIBs. The data suggested an increased risk of CHF with rofecoxib (RR 1.3) and a decreased risk of CHF with celecoxib (RR 0.6). The observation that use of DMARDs was associated with a reduction in the risk of hospitalisations for CHF in the RA cohort is consistent with the finding that tumour necrosis factor α is one of the principle inflammatory mediators of CHF.^44^ Also consistent with other observations is the increased risk with rofecoxib alongside a decreased risk with celecoxib, suggesting the absence of a class effect with respect to COXIBs for some types of cardiovascular morbidity.

## Conclusion

COXIBs appear to be prescribed preferentially to patients who were at an increased risk of cardiovascular events compared with patients prescribed nonspecific NSAIDs.^45^
